# The Role of Diet in Crohn’s Disease: From Etiology to Evidence-Based Management

**DOI:** 10.7759/cureus.86891

**Published:** 2025-06-27

**Authors:** Fares Jamal, Mayar H Alatout, Muhammad Ali Khan, Syed B Pasha, Talha A Malik

**Affiliations:** 1 Hematology and Oncology, Mayo Clinic, Phoenix, USA; 2 Colorectal Surgery, Mayo Clinic, Phoenix, USA; 3 Gastroenterology and Hepatology/Transplant Nephrology, Mayo Clinic, Phoenix, USA; 4 Gastroenterology, University of Pittsburgh Medical Center, Western Maryland, Cumberland, USA; 5 Gastroenterology and Hepatology, Mayo Clinic, Phoenix, USA

**Keywords:** crohn’s disease, crohn’s disease exclusion diet (cded), diet, exclusive enteral nutrition (een), inflammatory bowel disease (ibd), malnutrition., mediterranean diet

## Abstract

Crohn’s disease (CD) is a chronic, relapsing inflammatory bowel disease increasingly linked to environmental and dietary factors. This narrative review explores the role of diet in the pathogenesis and management of CD across its clinical spectrum. Emerging evidence implicates Westernized dietary patterns, characterized by high intakes of ultra-processed foods, saturated fats, and low fiber, contributes to intestinal dysbiosis, barrier dysfunction, and inflammation in genetically susceptible individuals. However, dietary patterns rich in minimally processed, plant-based foods, such as the Mediterranean diet, are associated with anti-inflammatory benefits. Nutritional therapies, including exclusive enteral nutrition (EEN) and the Crohn’s Disease Exclusion Diet (CDED), have shown efficacy in inducing and maintaining remission, particularly in pediatric populations. The role of specific macronutrients, food additives, and emulsifiers in modulating gut inflammation and microbiota composition is also reviewed. Furthermore, the importance of early malnutrition screening, micronutrient assessment, and individualized dietary counseling is emphasized. Integrating dietary strategies into multidisciplinary CD management may improve clinical outcomes, enhance quality of life, and reduce long-term disease burden. Future research should focus on personalized nutrition approaches and tools to support adherence and sustainability.

## Introduction and background

Crohn’s disease (CD), one of the two principal phenotypes of inflammatory bowel disease (IBD), is a chronic, relapsing, immune-mediated disorder of the gastrointestinal tract [1]. Its clinical course is heterogeneous, and, depending on the genotypic and phenotypic profile of each patient, it shows variable responses to therapy [2-4]. With about 1.5 million people affected in the United States and two million in Europe, IBD has long been viewed as a condition of highly industrialized nations [5-8]. Recent epidemiological data, however, reveal a rising incidence on every continent, particularly in newly industrialized regions whose populations are rapidly adopting Western lifestyles [9-11]. While incidence appears to be stabilizing in many high-income countries, prevalence continues to climb because patients are diagnosed young and rarely die of the disease, and modeling studies predict that several high-income regions may exceed a prevalence of 1% within the next decade [12]. As incidence accelerates in Asia, Africa, and Latin America, and prevalence compounds in North America and Europe, the global burden of IBD has already surpassed 0.3% [6,10,13-22].

The changing epidemiology highlights the importance of environmental influences that interact with fixed genetic risk. Among many external factors studied, diet commands particular interest because it is universal, modifiable, and in direct contact with the intestinal mucosa. Contemporary dietary shifts feature higher intakes of animal protein, saturated fats, refined sugars, and ultra-processed foods (UPFs), alongside lower consumption of fiber-rich fruits, vegetables, and whole grains [23]. 

Interventional evidence is growing. Exclusive enteral nutrition (EEN) can induce remission in pediatric CD and has long been the first-line therapy in many centers. More recently, the Crohn’s Disease Exclusion Diet (CDED) combined with partial enteral nutrition has shown superior tolerability and comparable efficacy, with sustained remission accompanied by favorable microbiome remodeling in randomized controlled trials [24,25]. A modified version of this diet continues to maintain remission in adults in real-world cohorts [26]. Such findings reinforce the potential for structured dietary interventions, both to prevent relapse and to serve as adjuncts or alternatives to pharmacotherapy. 

Given the escalating prevalence of CD worldwide, its profound impact on quality of life and healthcare costs, and the expanding body of evidence linking dietary patterns with disease onset and course, a comprehensive appraisal of diet in CD is timely. Clarifying these relationships will support personalized nutritional counseling, inform integration of diet into therapeutic algorithms, and guide public health strategies aimed at primary prevention. This review examines the role of diet in both the pathogenesis and management of CD. 

## Review

Methods

We conducted a narrative literature search to identify publications that examine the relationship between diet and CD pathophysiology or management. We chose a narrative review format to synthesize the broad and heterogeneous literature on diet in CD, which spans diverse dietary interventions, study designs, and outcome measures. This approach allowed us to integrate mechanistic insights with clinical findings, highlight emerging patterns, and identify gaps in the evidence that would not be adequately captured by a systematic or scoping review. PubMed, Embase, Web of Science, and Scopus databases were queried from inception to April 2025, using combinations of Medical Subject Headings (MeSH) and free-text terms for Crohn’s disease, inflammatory bowel disease, diet, dietary patterns, nutrition, food components, and specific nutrients. Reference lists of key articles and recent reviews were also manually searched to capture additional studies. Peer-reviewed articles published in English that reported original clinical, epidemiologic, interventional, or mechanistic data, as well as systematic reviews relevant to dietary exposures or interventions in CD, were included. Single-patient case reports, conference abstracts without full manuscripts, and non-English papers were excluded. Discrepancies in study selection were resolved through discussion. For each eligible study, we extracted design, population characteristics, dietary assessment methods, main outcomes, and key findings. Results were synthesized narratively to highlight consistent themes, emerging mechanisms, and areas of uncertainty. 

Pathogenesis of Crohn’s disease 

CD is a multifactorial disorder arising from a complex interplay of genetic, immunological, environmental, and microbial factors [27-29]. An integral feature of its pathogenesis is a dysregulated immune response, involving both innate and adaptive mechanisms that fail to maintain tolerance to intestinal antigens. This dysfunction results in excessive activation of intestinal macrophages, neutrophils, and helper T-cells, particularly Th1 and Th17 subsets, which produce proinflammatory cytokines such as tumor necrosis factor alpha (TNF-α), interferon-γ, interleukin 12 (IL-12), IL-17, and IL-23, perpetuating chronic mucosal and transmural inflammation [27,30]. Impaired recognition and clearance of bacterial components, in part due to defective nuclear factor kappa-light-chain-enhancer of activated B cells (NF-κB) signalling, lead to increased bacterial persistence and sustained immune activation [31]. Regulatory mechanisms are further compromised by inadequate T regulatory cell function and diminished production of immunosuppressive cytokines like IL-10 and transforming growth factor beta (TGF-β), and this contributes to the persistent inflammation in CD patients [30]. 

Genetics is a pivotal factor in the development of CD. This is evidenced by a significantly increased risk among first-degree relatives and the identification of over 200 genetic risk loci through genome-wide association studies (GWAS) [32]. Siblings of affected individuals have 35 times more risk of developing the disease than the general population [33]. Nucleotide-binding oligomerization domain-containing protein 2 (NOD2) is identified as the most important genetic factor influencing CD [34]. NOD2 is critical in bacterial sensing, and ATG16L1 is involved in autophagy, and together they are strongly linked to CD susceptibility [35]. A study by Cleynen et al. suggests that genetic profiling, particularly involving NOD2 and other loci such as IL23R and Janus kinase 2 (JAK2), has shown potential for stratifying patients by risk of severe disease phenotypes and may guide early, targeted therapeutic interventions, though further validation is required before widespread clinical implementation [28]. 

The likelihood of developing CD, as well as its progression, is also heavily influenced by environmental exposures. Smoking is the strongest, most well-studied modifiable risk factor, increasing the risk of developing CD and is associated with more severe disease and higher relapse rates [36]. Nicotine and other smoke constituents induce oxidative stress, impair mucosal healing, alter the gut microbiota, and promote intestinal fibrosis [37]. Other environmental influences include chronic exposure to air pollutants (such as nitric dioxide and carbon monoxide), which is associated with increased IBD incidence and hospitalizations, likely through mechanisms involving oxidative stress and immune dysregulation [38]. Furthermore, dietary patterns, particularly Western diet, can disrupt gut microbiota and barrier function, promoting further inflammation. 

The role of diet in the development and progression of Crohn’s disease 

While its exact etiology is multifactorial, diet has long been implicated as a key environmental factor influencing both the risk of developing CD and the exacerbation of symptoms in those affected. In recent years, research has increasingly focused on how specific foods, nutrients, additives, and overall dietary patterns may trigger the onset of CD or precipitate disease flares. Understanding these dietary triggers is crucial, as diet is a modifiable factor that could be targeted for prevention and management strategies. 

A Westernized diet, typically high in animal fats, red and processed meats, and refined sugars, and low in fiber-rich plant foods, has been consistently associated with an increased risk of developing CD. An umbrella review of dietary studies concluded that high intake of red and processed meats, other processed foods, and sugary foods correlates with a higher incidence of IBD, including CD. In parallel, insufficient consumption of protective foods like vegetables, fruits, and dietary fiber is linked to greater IBD risk [39]. Together, these findings suggest that unbalanced eating patterns may initiate the pathogenic processes of CD. 

These associations highlight the complex and multifactorial role of diet in CD, where the cumulative impact of long-term dietary habits, rather than single foods, appears to shape disease risk [40,41]. Evidence from birth cohort and maternal-infant studies indicates that diet during infancy and early childhood, as well as the duration of exposure to Western eating patterns, influences later susceptibility [42,43]. At the population level, the global shift toward highly processed, calorie-dense diets has paralleled the rising incidence of IBD in regions undergoing rapid urbanization and economic transition [44,45]. In addition, widely used food additives such as emulsifiers can disturb host-microbe interactions and provoke intestinal inflammation, further supporting a causal role for diet in CD pathogenesis [46,47]. Given this multifaceted influence, a detailed examination of individual dietary constituents, including macronutrients, additives, and processing methods, is essential to clarify their specific contributions to CD pathogenesis and to inform evidence-based prevention and management strategies. 

Ultra-processed food 

Developed at the Center for Epidemiological Studies in Health and Nutrition, University of São Paulo, Brazil, the NOVA system groups foods “according to the extent and purpose of the processing they undergo” [48] (Table 1). It defines food processing as the physical, biological, and chemical processes that occur after foods are separated from nature and before they are consumed or used in dishes and meals [49,50]. UPFs occupy the most industrialized tier; they are formulations of inexpensive substrates, cosmetic additives, flavorings, and texturizers designed for palatability, shelf life, and convenience. In its landmark 2019 report, the Food and Agriculture Organization of the United Nations (FAO) warned that diets dominated by UPFs displace fiber- and micronutrient-rich staples, increase free sugars and saturated fat, and are consistently linked with non-communicable diseases, urging a reorientation of dietary guidelines toward minimally processed foods [51]. Mechanistic data reviewed in 2024 highlights how emulsifiers (polysorbate-80, carboxymethyl cellulose), maltodextrins, and synthetic sweeteners alter the mucus barrier, thin tight-junction complexes, favor bile-tolerant pathobionts, and amplify innate immune signaling, changes that mirror early mucosal lesions in CD [52]. 

**Table 1:** NOVA Food Classification System: Categories and Examples Information compiled from references [48-50]

NOVA Group	Short Name	What It Is / Key Features	Everyday Examples
NOVA 1	Unprocessed or Minimally Processed	Edible parts of plants, animals, mushrooms, or algae that are fresh, chilled, frozen, dried, ground, or fermented without added substances.	Apples, fresh meat, plain yogurt, dry beans, frozen spinach
NOVA 2	Processed Culinary Ingredients	Substances extracted from NOVA 1 foods or nature and used in kitchens to season or cook. Usually consumed in small amounts.	Vegetable oil, butter, sugar, honey, salt, starches
NOVA 3	Processed Foods	Products made by adding NOVA 2 ingredients to NOVA 1 foods to increase shelf-life or improve flavor/texture; contain 2–3 ingredients.	Canned vegetables with brine, cheese, fresh bread, jam
NOVA 4	Ultra-Processed Foods	Industrial formulations with little or no intact NOVA 1 food, plus additives for color, flavor, texture, or shelf life; ready-to-eat or heat.	Soft drinks, packaged cookies, instant noodles, chicken nuggets, flavored chips

In the PURE study, which followed 116,087 adults across 21 countries for a median of 9.7 years, consuming five or more UPF servings per day conferred an 82% higher hazard of developing IBD, with comparable risk estimates for CD and ulcerative colitis (UC) [53]. The association appears stronger and more specific for CD in Western cohorts. In a cohort of over 185,000 UK Biobank participants, those in the highest quintile of UPF intake had a twofold increased risk of developing CD, but no significant increase in risk for UC; moreover, among prevalent IBD cases, higher UPF intake predicted a fourfold greater need for surgery [54]. A 2023 systematic review and meta-analysis integrating seven cohorts (n=2.6 million) confirmed a dose-response relationship, estimating a 29% rise in CD risk for each 10 % increment in calories from UPFs [55].

 UPFs owe their shelf life, texture, and visual appeal to a small group of ingredients like emulsifiers, non-nutritive sweeteners, synthetic colors/flavors, and stabilizers that rarely appear in home cooking. A recent review concluded that these additives, rather than macronutrient composition per se, are the common thread linking high-UPF diets to CD through effects on the microbiome, mucus barrier, and innate immunity [52]. Mouse and in vitro human microbiome models show that emulsifiers like carboxymethyl cellulose (CMC) and polysorbate-80 (P80) erode the inner mucus layer, pull bacteria toward the epithelium, and raise mucosal lipopolysaccharide, collectively priming low-grade colitis [56]. Owing to the increasing prevalence of obesity and type 2 diabetes mellitus, many people have been advised to transition from sugar to artificial sweeteners [57]. Hetta et al. synthesized 30 rodent and five human trials and concluded that non-nutritive sweeteners consistently disturb microbiota diversity and reduce short-chain fatty acid producers [58]. Parallel work links sucralose or Splenda® to Proteobacteria blooms, myeloperoxidase activation, and worse ileitis in IL-10-knock-out and SAMP1/YitFc models that simulate CD [59,60]. Some studies have not found inflammation-related microbiome changes. In a study using C57BL/6J mice, sucralose at 0.72 mg/ml, equivalent to the European Food Safety Authority (EFSA)’s acceptable daily intake of 15 mg/kg after metabolic adjustment, showed no consistent effects on gut microbiota [61]. Artificial flavors and colors were also linked to gut inflammation in many studies [62-64]. 

Until larger trials stratified by genetics, microbiome state, and additive subtype emerge, clinicians may reasonably recommend limiting brightly colored confectionery, artificially sweetened beverages, and packaged foods rich in emulsifiers, especially for young or genetically susceptible CD patients, while acknowledging that rigorous proof of causation remains a work in progress. 

Fats

High dietary fat, particularly the saturated fats and omega-6 (ω-6) polyunsaturated fatty acids (PUFAs) that dominate Western eating patterns, has long been linked to CD. One of the earliest signals came from a 1966-1985 Japanese survey in which newly diagnosed patients reported far higher total fat intake than population controls [65]. Mechanistic work suggests multiple, complementary routes to injury. Saturated fats activate NF-κB in adipocytes and macrophages, loosen tight junctions, and foster an IBD-like dysbiosis [66-68]. 

Dietary fats are metabolized into omega-3 (ω-3) (α-linolenic acid, eicosapentaenoic acid (EPA), docosahexaenoic acid [DHA]) and ω-6 (linoleic acid [LA], arachidonic acid [AA]) families [69]. The balance between ω-6 and ω-3 PUFAs shapes the basal inflammatory tone of the intestinal mucosa and has therefore been scrutinized in CD. Epidemiologic transition studies show that pre-industrial diets delivered an almost 1:1 ω-6 to ω-3 ratio, whereas modern Western patterns approach, or exceed, 15:1 to 16.7:1 [70-72]. This shift parallels the global rise in CD incidence [73]. High dietary ω-6 fatty acids saturate cell membranes with arachidonic acid, crowding out ω-3 derived lipids that normally give rise to pro-resolving mediators such as resolvins, protectins, and maresins [74]. The arachidonic acid surplus then flows into cyclooxygenase and lipoxygenase pathways, amplifying production of proinflammatory eicosanoids: leukotriene B₄, thromboxane B₂, prostaglandin E₂, and other leukotrienes. These potent signals further prime the immune response by activating the costimulatory molecules on dendritic and T cells, thereby accelerating T-cell proliferation and sustaining mucosal inflammation [75-77]. 

A multicenter Canadian pediatric case-control study found that children in the top third of the dietary ω-6:ω-3 ratio were almost twice as likely to develop new CD, but only if they carried gene variants in fatty acid desaturase 2 (FADS2) and cytochrome P450 family 4 subfamily F member 3 (CYP4F3), two enzymes that guide PUFA metabolism [78]. This gene-diet link shows that an unbalanced ω-6:ω-3 ratio is most harmful when a child’s own ability to convert α-linolenic acid (ALA) into EPA and DHA is genetically weak. 

In a Mendelian randomization study of 17 circulating fatty-acid metrics, Zhou and Zhou showed that higher DHA levels and higher ω-3-to-total-fat ratios were significantly protective for IBD, most strongly for CD (OR ≈ 0.79, FDR = 0.04) [79]. Conversely, animal work reinforces harm from excess ω-6 and saturated fat: IL-10-knockout mice fed high-ω-6 diets develop more severe colitis, and saturated or long-chain fats intensify Th17 and CD3⁺ T-cell infiltration while depleting regulatory T cells [80-83]. Oxidized lipids further activate natural killer cells to release cytotoxic enzymes, and epithelial cells respond by secreting neutrophil-recruiting chemokines [84]. 

Yet large prospective cohorts and rigorous evidence syntheses warn that the picture is not uniform. Across ~200,000 women who were followed for 26 years in the Nurses’ Health Study (NHS) I and II, neither total dietary ω-6 PUFA nor the ω-6: ω-3 ratio predicted incident CD after full adjustment (hazard ratio (HR) ≈ 0.95, p = 0.41) [85]. Likewise, a Cochrane systematic review of 83 randomized trials judged the benefits of increasing ω-3 (typically fish oil) or altering ω-6 intake to be inconclusive for CD relapse or biomarker modulation [86]. Most other human case-control and cohort studies, except for one, have similarly found no significant association between total fat, PUFA, or ω-6 load and CD risk [87-93]. 

Like all other macronutrients, dietary fats are also reported to contribute to CD pathogenesis mediated via dysbiosis. Several studies have shown dramatic changes in gut flora, consistent with IBD-associated changes, in mice fed a high-fat diet [81,94-97]. Even saturated fats from milk have been reported to produce this pattern of dysbiosis, even though consumption of milk has overall demonstrated a CD protective effect in prospective human studies [97,98].

Together, epidemiologic, genetic, and experimental data converge on a model in which excessive ω-6 and saturated-fat exposure tilts mucosal immunity toward chronic inflammation, particularly in genetically or microbially susceptible hosts, while abundant ω-3 (especially DHA) can temper that response. However, the magnitude and consistency of these effects vary, underlining the need for personalized nutritional and genomic approaches in CD prevention and management. 

Carbohydrates 

Rapidly digestible and highly refined carbohydrates, whether delivered as table sugar, polished grains, or sugar-sweetened beverages, emerge as a distinct dietary risk axis for CD. Genome-wide association studies and functional analyses converge on a model in which excess simple sugars shift the gut microbiome toward mucin-degrading taxa, thin the mucus layer, upregulate toll-like-receptor signaling, and loosen epithelial tight junctions, events that prime innate and adaptive immunity for chronic inflammation [99]. In the United Kingdom Biobank cohort of 121,490 adults followed for a decade, drinking more than one sugar-sweetened beverage per day doubled the hazard of incident CD, with no association for artificially sweetened drinks or natural juices [100]. A separate multinational study that tracked diet quality scores found that patterns high in refined sugars and low in whole grains predicted new-onset CD even after adjusting for body mass index and smoking [101]. In the pediatric population, Thomas et al. noticed that CD patients consumed more bakery sweets and fewer vegetables compared to their siblings [102]. However, mixed results were seen when the role of soft drinks in the development of CD was evaluated [103]. Furthermore, by increasing bile output, refined sugars also demonstrated overgrowth of opportunistic bacteria such as *Clostridium difficile* and *Clostridium perfringens* in the gut [104,105]. 

Carbohydrate quality continues to matter after diagnosis. In a survey-based IBD cohort, Crohn’s patients who avoided high-fiber foods were nearly 60% more likely to enter symptomatic flare during the ensuing six months than those who continued to eat fiber liberally [106]. Conversely, highly fermentable short-chain carbohydrates can aggravate gas and pain even when inflammation is quiescent. A 2023 systematic review of randomized trials concluded that a low-FODMAP (fermentable oligosaccharides, disaccharides, monosaccharides, and polyols) strategy reduced abdominal pain scores in CD [107]. 

Even a short burst of dietary sugar can sensitize the gut to injury. In a mouse dextran sulfate sodium (DSS)-colitis model, just two days on a high sugar diet reshaped the microbiota, drained acetate and butyrate pools, and left the animals far more vulnerable to chemically triggered colitis [108]. 

A contradictory narrative also exists. Two large prospective studies from the United States, NHS I and II, have not reported carbohydrate associations for CD to date [109]. The European Prospective Investigation and Nutrition in Cancer (EPIC) study, which also investigated the role of sugars and starch in CD, could not find any significant association either [110]. One interesting finding from the EPIC-IBD sub-cohort, however, was a decreased risk of CD in participants with higher milk consumption, irrespective of total calcium and dairy product intake in a dose-independent fashion [98]. 

Overall, the association between carbohydrates and CD appears to be inconsistent and weak. This is because most of the evidence comes from animal studies. Despite this, the role of carbohydrates needs further investigation before any definite claims can be made. 

Proteins 

Compared to carbohydrates, higher protein intake, especially from animal sources such as meat, has shown a stronger link with increased risk of IBD [98]. In the NHS I and II cohorts, which included 260 CD and 319 UC patients, greater consumption of red meat and dietary heme iron was associated with a higher risk of UC. This relationship was also influenced by genetic factors. A coding variant, rs1801274, in the *FCGR2A* gene, which is involved in humoral immunity, was associated with anywhere from a reduced to a threefold higher risk of UC, depending on the individual’s genotype [109,111,112]. Similar associations between red meat and UC were seen in other large cohort studies like EPIC and E3N, although this pattern was not observed in CD [87,90,108,111,113]. 

High-protein diets can lead to changes in the gut microbiome. These include increased activity of bacterial enzymes such as beta-glucuronidase, azoreductase, and nitroreductase, which are known to promote inflammation [114]. A meat-rich diet is also linked to the overgrowth of sulfate-reducing bacteria and higher levels of hydrogen sulfide, a substance toxic to colon cells and linked to immune system problems [115]. This microbial shift has been associated with UC but not clearly with CD [116]. Animal studies have found that diets high in red meat can raise levels of inflammatory molecules like sphingosine-1-phosphate and worsen colitis symptoms triggered by dextran sodium sulfate [117,118]. Another study in mice showed that a high-casein diet caused changes in the gut microbiome that resemble those seen in IBD [119]. 

In Japan, increasing meat consumption has been linked with a rising incidence of CD [120]. Although several studies have examined whether a high total protein intake increases CD risk, most have not found a strong association [89,91,92,121,122]. Some of these studies did show a possible link with meat, but not with other protein sources like dairy and eggs [89,90,122,123]. Among different types of meat, red meat was the only one consistently associated with CD, while results for fish were inconsistent [89,122,124].

The differences in findings between meat and other protein sources suggest that other components in meat might contribute to CD risk. Along with protein, red meat contains high amounts of saturated fats, and its intake may reflect a generally high-fat diet [125,126]. White meat, which is lower in fat, was associated with a lower risk of IBD in at least one study [127]. Processed meats, which often contain harmful substances like heterocyclic amines and polycyclic aromatic hydrocarbons, have also been linked to a greater risk of IBD [128]. Another theory, known as the cold-chain hypothesis, proposes that meat may carry high levels of Yersinia bacteria, which could play a role in the development of CD [129]. 

Dietary management strategies across the Crohn’s spectrum

Mediterranean Diet 

The Mediterranean diet is recommended for patients with CD [130]. It emphasizes a high intake of vegetables, fruits, nuts, legumes, whole grains, seafood, and olive oil; modest intake of fish; and limited consumption of UPFs and red meat [131,132]. This composition makes it a diet rich in fiber and low in saturated fat. Olive oil, the primary fat source in the Mediterranean diet, is known for its protective effects against autoimmune, inflammatory, and cardiovascular diseases [133]. 

Unlike the Western diet, which is largely based on animal protein, the Mediterranean diet primarily incorporates plant-based protein [134]. Plant protein has been shown to reduce inflammation in CD, whereas animal protein has been associated with increased disease activity and symptom exacerbation [134,135]. The American Gastroenterology Association (AGA) Clinical Practice Update recommends the Mediterranean diet for all CD patients unless contraindicated [130]. The Mediterranean diet is preferred over other dietary approaches due to its broad health benefits and high adherence rates [136]. 

Several studies have demonstrated the association between adherence to the Mediterranean diet and decreased CD activity. For instance, Papada et al. found that disease activity and inflammation significantly declined in CD patients who followed the Mediterranean diet [137]. Similarly, Chicco et al. reported normalization of C-reactive protein (CRP) and fecal calprotectin (FCP) levels in patients adhering to the Mediterranean diet [138]. These findings are supported by additional studies demonstrating the Mediterranean diet’s role in controlling inflammation and normalizing CRP and FCP levels [139]. A meta-analysis by Limketkai et al. showed a strong association between high intake of fiber, an essential component of the Mediterranean diet, with improved quality of life and slower disease progression in CD patients [140]. Migdanas et al. also observed a positive correlation between adherence to the Mediterranean diet and improved quality of life [141]. In summary, these findings highlight the role of the Mediterranean diet in reducing inflammation and enhancing the quality of life in CD patients. 

However, there are certain limitations in implementing the Mediterranean diet in the management of CD. Many healthcare professionals and patients lack a clear understanding of the diet’s composition [139]. Furthermore, some patients may be hesitant to adopt a diet that diverges from their cultural food preferences [142]. The availability of certain Mediterranean diet ingredients, particularly fresh fruits and vegetables, may also be limited in colder climates [143]. To address these barriers, educational resources should be available for practitioners and patients to explain the diet’s components, provide seasonal ingredient guides, and offer culturally adapted versions of the Mediterranean diet. Further research on the use of frozen fruits and vegetables may help make the Mediterranean diet more accessible and practical year-round. 

Exclusive Enteral Nutrition (EEN)

Enteral Nutrition is a dietary intervention in which all caloric needs are met through a nutritionally complete liquid formula, with complete exclusion of solid food. The formula can be administered either orally or via a feeding tube, with both routes demonstrating comparable efficacy [144]. EEN is typically prescribed for six to eight weeks, after which patients gradually transition back to a regular diet [145]. EEN formulas are classified based on their nitrogen source: elemental (antigen-free, composed of free amino acids), semi-elemental (containing oligopeptides), and polymeric (based on whole proteins) [146]. 

The European Society for Pediatric Gastroenterology, Hepatology and Nutrition (ESPGHAN), European Crohn’s and Colitis Organization (ECCO), AGA, and the North American Society for Pediatric Gastroenterology, Hepatology and Nutrition (NASPGHAN) recommend EEN as the first-line therapy for inducing remission in pediatric CD [130,146]. 

The beneficial effects of EEN are thought to arise from multiple mechanisms. The exclusion of certain dietary components reduces inflammation and promotes mucosal healing [147]. EEN has been shown to lower the number of cytokine-producing cells and increase regulatory T cells in the intestinal mucosa of CD patients [148-150]. This has been witnessed by the decrease in inflammatory markers following the use of EEN in children [151]. Furthermore, Leache et al. and Schwerd et al. demonstrated a decline in bacterial diversity, especially the Bacteroides group, which leads to a decrease in inflammation [150,152]. 

Numerous studies have compared EEN to corticosteroids for remission induction, particularly in the pediatric population, demonstrating promising results of EEN compared to corticosteroids. EEN often demonstrates equal or superior outcomes in children and is preferred unless contraindicated or ineffective [153]. Compared to corticosteroids, EEN is associated with superior mucosal healing, favorable shifts in gut microbiome, better weight gain, improvement in vitamin D levels and bone metabolism, an early rise in insulin-like growth factor 1, and enhanced quality of life [154-160]. In a study by Guo et al., four weeks of EEN led to significant improvements in all domains of the Inflammatory Bowel Disease Questionnaire (IBDQ), including bowel symptoms, emotional status, social functioning, and systemic symptoms [161]. Similar findings were reported by Sharma et al. [162]. Moreover, preoperative use of EEN has been linked to reduced postoperative complications [163]. However, corticosteroids remain more effective than EEN in adult populations [164]. 

The primary limitation of EEN is poor adherence, which can stem from various factors. The most frequently cited barrier is its unpleasant taste, with reported intolerance ranging from 0% to 41% [165]. Additional challenges include a lack of support and low motivation to complete the regimen. Nonetheless, adherence rates are generally higher in children than in adults [166]. 

*Crohn’s Disease Exclusion Diet (CDED)* 

The CDED is a structured dietary approach that combines whole foods with partial enteral nutrition, typically using a specific formula like Modulen® IBD Powder (Nestlé S.A., Vevey, Switzerland) [167]. Its goal is to reduce exposure to dietary components that may impair gut immune function, compromise the intestinal barrier, and disrupt the microbiome [146]. 

The CDED, combined with partial enteral nutrition, is structured in three phases. In Phase 1 (weeks 1-6), patients consume mandatory foods like fish, chicken, and eggs, with allowed items such as rice, tomatoes, and olive oil, while limiting certain fruits and vegetables. Enteral formula provides 50% of the energy needs. Phase 2 (weeks 7-12) expands the diet to include foods like tuna, whole-grain bread, oats, and some legumes, with formula reduced to 25%. In the Maintenance Phase (week 13 onward), the diet becomes more flexible, adding seafood, grains, coffee, some dairy, and limited alcohol, while still avoiding UPFs [130,168]. 

AGA recommends considering CDED in patients who cannot tolerate enteral nutrition or who have not responded adequately to biologic therapy [130]. The diet aims to promote mucosal healing by restoring barrier integrity and reducing inflammation [169]. 

Evidence from multiple studies supports its effectiveness. In a 12-week trial, Sigall-Boneh et al. reported an 84% remission rate with CDED plus partial enteral nutrition [167]. Similar results were seen by Szczubelek et al. in 2021, with an 82.1% remission rate [170]. Yanai et al. demonstrated sustained remission at 24 weeks [171]. Notably, comparisons of CDED with and without partial enteral nutrition revealed no significant differences in remission rates, raising questions about the necessity of the formula component [167,171]. Moreover, CDED, with or without partial enteral nutrition, led to remission in 61.9% of patients who had lost response to biologics [172]. 

Biomarker improvements, including reductions in FCP and CRP, have been documented in studies by Levine et al. [24], Szczubelek et al. [170], Yanai et al. [171], and Urlep et al. [173].

Despite these benefits, CDED has limitations. Adherence can be challenging due to the strict dietary requirements and need for careful meal planning [146]. Additionally, if not properly supervised, the diet may pose a risk for nutritional deficiencies [130]. 

Parenteral Nutrition

Parenteral nutrition is a way of delivering essential nutrients, like carbohydrates (as dextrose), proteins (as amino acids), and fats, directly into the bloodstream through a central or peripheral vein [174,175]. It also includes vital vitamins, minerals, and electrolytes. In CD, especially during active inflammation, patients often require more protein than usual, around 1.2-1.5 g/kg/day, and up to 2.5 g/kg/day after surgery for healing wounds or in cases of fistulas [175]. Some patients may need fat alternatives due to allergies; for example, switching to blends of olive oil, fish oil, soybean oil, or medium-chain triglycerides can help manage egg allergies [175]. 

While enteral nutrition is always preferred, when possible, parenteral nutrition becomes necessary when patients cannot meet their nutritional needs by mouth or through the gut for 7-10 days [174]. According to European Society for Clinical Nutrition and Metabolism (ESPEN) guidelines, parenteral nutrition is recommended in cases like bowel ischemia, high-output fistulas, severe bleeding, anastomotic leaks, bowel obstructions, or short bowel syndrome [176,177]. In some situations, parenteral nutrition can be combined with enteral feeding to ensure adequate intake [178]. 

The location of a bowel obstruction can influence whether parenteral nutrition is required. If the obstruction is lower in the intestine, placing an enteral feeding tube might not be feasible, making parenteral nutrition necessary. But if the blockage is higher up, it’s often worth trying enteral feeding first, with parenteral nutrition used only if that fails [178]. Similarly, only patients with prolonged ileus lasting over a week usually need parenteral nutrition [179]. Fistula output and location also matter; those with high-output (over 500 mL/day) or upper GI tract fistulas often need parenteral nutrition either alone or in combination with other nutrition [130,178,180]. 

The length of time a patient stays on parenteral nutrition depends on the reason for starting it. Sometimes, it’s used short-term, just a few days or weeks, to support nutrition before surgery. In other cases, like with ongoing high ostomy output or severe strictures, parenteral nutrition might be needed for a much longer period. When considering weaning, clinicians look at factors like oral intake, hydration status, lab values, and inflammation markers [175]. Patients who need less than a liter of parenteral nutrition per day often have a better chance of being weaned off [181]. If weaning doesn’t work, therapies like teduglutide (a GLP-2 analog) may help by improving small bowel function and water absorption [176,182]. However, this is usually only considered at least six months after the last bowel resection surgery [183]. 

Despite its benefits, parenteral nutrition carries risks. Complications generally fall into two categories: catheter-related and nutrition-related. Catheter-related complications include familiar risks like infections, blood clots, and air embolism [184]. Nutrition-related complications involve metabolic issues like blood sugar problems, electrolyte imbalances, liver dysfunction, or high triglyceride levels [185,186]. One serious concern is refeeding syndrome, which is why parenteral nutrition should be started in the hospital under the care of a specialized team. For patients continuing parenteral nutrition at home, regular lab monitoring, often weekly, is essential to adjust the nutrient formula as needed [175].

Table 2 compares different dietary therapies in CD.

**Table 2:** Comparison of nutritional and dietary therapies in Crohn’s disease CD: Crohn’s disease; GI: gastrointestinal; IV: intravenous References: [131,139,146,166,168,171,175,176]

Feature	Exclusive Enteral Nutrition	Crohn’s Disease Exclusion Diet	Mediterranean Diet	Parenteral Nutrition
Key features	100% liquid formula; excludes whole foods	Whole food-based; excludes additives, gluten, processed food; partial formula support	Rich in fruits, vegetables, fish, olive oil; low in red meat	100% IV, bypassing the GI tract entirely
Mechanism	Microbiota modulation, antigen exclusion, mucosal healing	Excludes dietary triggers while preserving diversity	Anti-inflammatory nutrients, fiber, polyphenols	Gut rest, complete nutritional support via IV
Tolerability/adherence	Often poor in adults; better in children	Higher adherence due to partial food intake	High adherence, lifestyle compatible	Requires central line; risk of infection, liver dysfunction
Duration of use	6–8 weeks for induction, then transition	Initial 6–12 weeks, followed by partial maintenance	Long-term, sustainable pattern	Short to medium term; often used as bridge to enteral or surgical therapy
Role in different CD phenotypes	First-line in pediatric inflammatory CD; bridge to surgery in adults	Non-stricturing CD; also used in maintenance	Adjunct in mild/moderate inflammatory CD	Penetrating disease, short bowel, severe strictures, high-output fistulas
Limitations	Monotony, cost, psychosocial barriers	Labor-intensive prep; requires patient education	Requires individualization in stricturing CD	Invasive, costly, requires monitoring and access expertise

Crohn’s disease phenotypes

**Figure 1:**
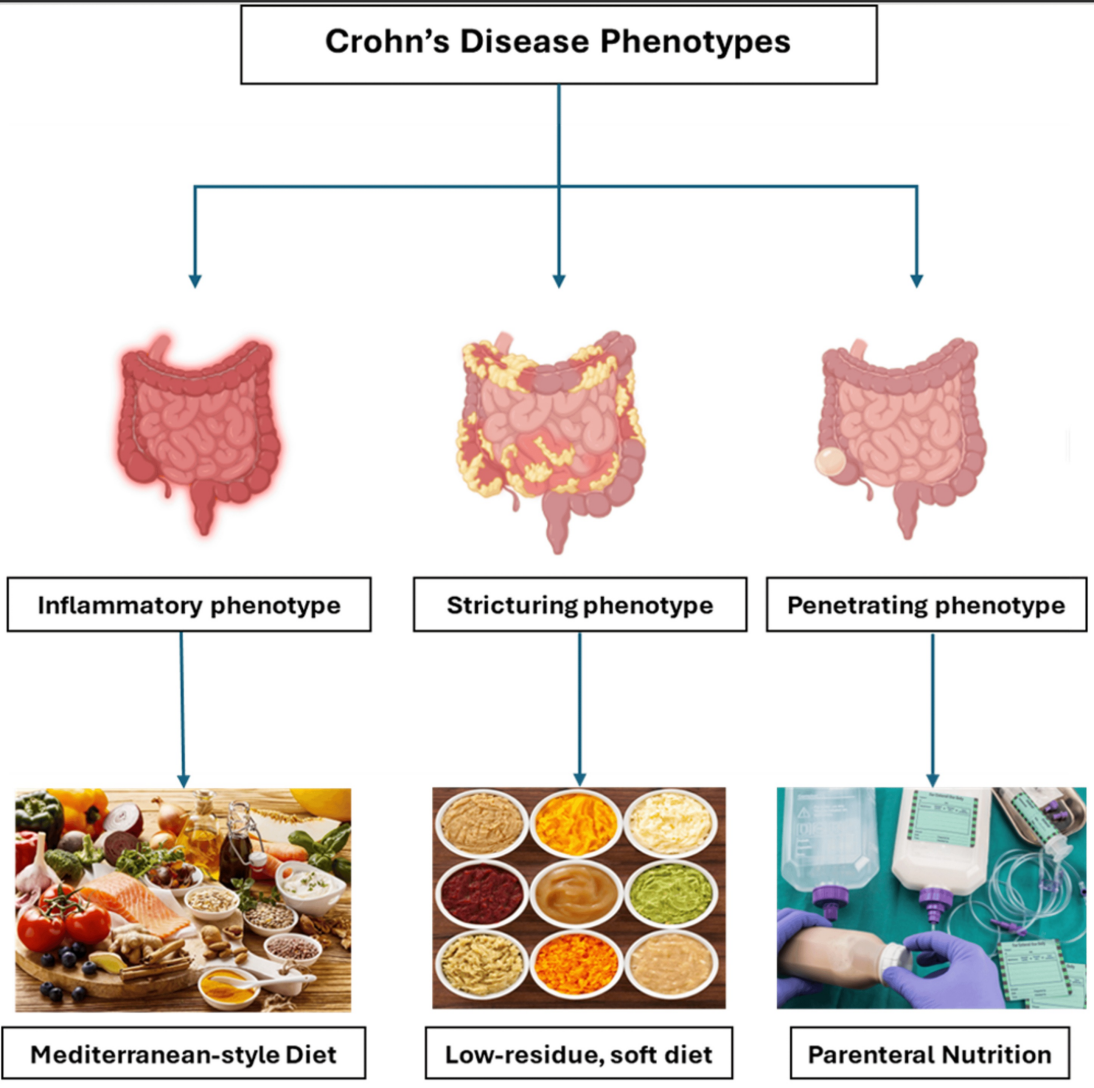
Dietary management strategies based on Crohn’s disease phenotypes Image Credit: Authors

CD can manifest with inflammation, strictures, or fistulas anywhere along the gastrointestinal tract [187]. Each phenotype has different dietary recommendations (Figure 1).

Inflammatory Crohn’s disease without structures 

As discussed earlier, patients with inflammatory CD, without evidence of strictures, should follow the Mediterranean diet unless contraindicated [130]. A diet rich in processed foods and low in vegetables and fruits has been associated with increased risk of inflammatory CD [188]. 

Stricturing Crohn’s disease 

Strictures, a well-known complication of CD, are defined as narrowing in the gastrointestinal tract, affecting approximately 25% of patients in the small intestine and about 10% in the colon [189]. Several risk factors contribute to the formation of strictures, including age <40 years at diagnosis, steroids used to control flares, perianal disease, and a history of smoking [190]. CD patients with strictures can introduce plant-based foods and fiber into their diet after extensive chewing and cooking to soften the texture and reduce residue [130]. 

Penetrating Crohn’s disease 

Fistulas are abnormal tracts that form between the GI tract and nearby organs or the skin, often seen in patients with penetrating CD [187]. The risk of developing fistulas increases with disease duration, rising to approximately 50% after 20 years [191]. Diagnosed with CD at an age younger than 40 years or a history of strictures increases the risk of forming a fistula [192]. CD patients with high-output fistula (>500 ml/day) should follow a parenteral nutrition diet to increase the chances of fistula closure [193]. Similarly, CD patients with intra-abdominal abscess should follow the parenteral nutrition diet while waiting for surgery to decrease the inflammation and spread of infection [130]. 

Nutrition assessment and malnutrition in Crohn’s disease

Malnutrition is a prevalent concern in CD, with rates reaching 38.9% in patients in remission and increasing to 82.8% during active disease phases [194]. According to the Global Leadership Initiative on Malnutrition (GLIM), a diagnosis of malnutrition requires at least one etiologic criterion and at least one phenotypic criterion [195]. Etiological criteria include decreased food intake and disease inflammation or burden; moreover, phenotypic criteria include decreased muscle mass, low BMI, and involuntary weight loss [195]. 

Several tools are available to assess nutritional risk in CD. Among them, the Malnutrition Universal Screening Tool (MUST) and the Saskatchewan Inflammatory Bowel Disease-Nutrition Risk (SaskIBD-NR) screening tools had the highest sensitivity (100%) for detecting patients at moderate to high risk of malnutrition [196]. However, the sensitivity of all the tools decreased to detect malnutrition over a 6-12 month period; however, MUST and SaskIBD-NR remained the most sensitive tools. Additional screening tools include the Subjective Global Assessment (SGA) scale, the Nutritional Risk Index (NRI), Onodera’s Prognostic Nutritional Index (PNI), and Controlling Nutritional Status (CONUT) [194,197,198]. 

Multiple risk factors contribute to malnutrition in CD, including prior surgery related to CD, diarrhea, and active inflammation [196]. This could result from multiple factors, including reduced oral intake, avoidance of perceived trigger foods, medication side effects, malabsorption, fluid losses, altered GI anatomy from prior surgeries, and increased nutritional demand during active inflammation [196]. Malnutrition is associated with higher rates of surgical complications, suboptimal response to medical therapy, decreased quality of life, prolonged hospital stays, and more frequent disease flares [199-201].

Micronutrient deficiencies are common in patients with CD, with iron, vitamin B12, vitamin D, folic acid, selenium, and zinc being the most frequently affected [202]. These deficiencies may lead to significant comorbidities, including osteoporosis, anemia, carcinogenesis, poor wound healing, and chronic inflammation [203]. Furthermore, some of these deficiencies may lead to irreversible damage; for instance, vitamin B12 deficiency causes posterolateral cord syndrome, and copper deficiency leads to myelopathy [204,205]. Therefore, annual screening for micronutrient deficiencies is recommended [174]. 

Iron 

ESPEN recommends treating all CD patients with iron when iron deficiency anemia is diagnosed [206]. Oral iron is the preferred first-line treatment in patients with mild anemia, clinically inactive IBD, and no history of oral iron intolerance. Intravenous iron should be considered for those with active disease, prior intolerance to oral iron, hemoglobin levels below 10 g/dL, or those receiving erythropoiesis-stimulating agents [174,207]. 

Vitamin D 

ESPEN recommends monitoring serum 25-hydroxy vitamin D in patients with active CD, corticosteroid use, or with suspected vitamin D deficiency [206]. Ratajczak et al. suggest that patients with CD and vitamin D deficiency require higher supplementation doses than the general population [208]. Complications that could result from hypovitaminosis D, including osteopenia and osteoporosis, should be treated based on the current guidelines [174]. 

Magnesium 

There is no consensus regarding the magnesium level status in CD patients. However, when magnesium deficiency is detected, oral supplementation with magnesium is recommended [206]. 

Zinc 

Several studies demonstrated zinc deficiency in CD patients during the active and remission phases [209-211]. Low albumin levels during active disease may further predispose patients to zinc deficiency [212]. Oral zinc supplementation is recommended in CD patients with zinc deficiency [213]. 

*Selenium* 

According to Ringstad et al. [209], Wendland et al. [210], and Gentschew et al. [211], selenium levels are lower in CD patients compared to the general population, irrespective of disease phase. Oral supplementation is recommended when selenium deficiency is present [214]. 

*Folate* 

Folate deficiency is more common in CD patients with small bowel involvement or those treated with sulfasalazine [206]. Hence, patients with extensive small bowel resection, active disease, or patients on sulfasalazine should be treated prophylactically with folate supplements [215,216]. 

Cobalamin 

Vitamin B12 is absorbed in the ileum; hence, ileal resection secondary to CD may increase the susceptibility to vitamin B12 deficiency [217]. Supplementation should be considered in such patients to prevent neurologic complications [206]. 

Special clinical scenarios 

Managing CD during pregnancy and lactation requires careful balancing of disease control and fetal safety. Active disease at conception or during pregnancy is associated with adverse outcomes, including preterm birth and low birth weight [218]. Most maintenance therapies, including aminosalicylates, thiopurines, and anti-TNF agents, are considered safe during pregnancy and lactation, although methotrexate is contraindicated. Nutritional support is essential, as micronutrient deficiencies (iron, folate, vitamin D) are common and may impact maternal and fetal health [219]. 

Children and adolescents with CD frequently experience growth failure and delayed puberty, with up to 85% showing some degree of linear growth deficiency or pubertal delay at diagnosis [220]. Growth failure in pediatric patients is mainly due to chronic calorie insufficiency from poor intake and inflammation-driven anorexia, long-term corticosteroid use that suppresses growth hormone (GH) and insulin-like growth factor 1 (IGF-1) activity, and delayed puberty leading to sex hormone deficiencies that impair growth [220]. Early and aggressive control of inflammation, often with EEN or biologic therapy, is associated with improved growth outcomes. Regular monitoring of growth parameters and pubertal development is recommended, with timely intervention to address nutritional deficits and optimize bone health [221]. 

Extra-intestinal manifestations (EIMs) such as bone loss and non-alcoholic fatty liver disease (NAFLD) are increasingly recognized in CD. Chronic inflammation, corticosteroid use, and poor nutritional status contribute to reduced bone mineral density and increased fracture risk. A meta-analysis by Szafors et al. found a 38% increased overall fracture risk in IBD patients compared to healthy controls, and a more than twofold increase in vertebral fracture risk [222]. Adequate intake of calcium, vitamin D, and regular weight-bearing exercise are recommended for prevention. NAFLD is prevalent in Crohn’s patients, potentially exacerbated by high-fat diets and metabolic syndrome. Medical therapies, particularly corticosteroids and tofacitinib, can significantly alter lipid profiles, leading to elevated total cholesterol and low-density lipoprotein (LDL) levels, which may predispose patients to NAFLD. These metabolic changes are closely linked to systemic inflammation and are exacerbated by poor dietary habits and insulin resistance. Therefore, managing CD requires not only pharmacologic control of intestinal inflammation but also vigilant monitoring of metabolic health, emphasizing dietary modifications, regular exercise, and lipid management [223]. 

Patient-centered Implementation

Patient-centered care is a cornerstone of effective CD management, particularly due to the complex and preference-sensitive nature of treatment decisions. Shared decision-making empowers patients to participate in choosing therapies that align with their values and risk preferences. Using decision aids to present options in a patient-friendly way improves understanding, satisfaction, confidence, and increases treatment adherence. Tailoring the approach to individual needs enhances outcomes, reinforcing the value of personalized, collaborative care [224]. To further empower patients, culturally adapted meal plans and digital health tools can significantly benefit patients with CD. The AGA provides guidance on various dietary interventions for CD, including the Mediterranean diet, specific carbohydrate diet, and CDED [130]. These diets can be tailored to fit cultural preferences, enhancing adherence and effectiveness. For instance, the CDED has been successfully adapted across diverse ethnicities, emphasizing the importance of dietitians in customizing the diet to accommodate cultural behaviors and traditions [225]. Digital health tools, such as mobile apps and tele-nutrition, have shown promise in managing CD. A systematic review highlighted that digital health interventions, including mobile applications and telemedicine platforms, can effectively monitor disease activity and reduce healthcare utilization and costs [226]. These tools often include features for tracking symptoms, medication adherence, and providing educational resources, which can be particularly beneficial. They can enhance quality of life, treatment adherence, and medication management for patients with CD [227]. 

Future directions

Emerging evidence from recent studies explores how gut metabolome and microbiota signatures can predict response to enteral nutrition in children with active CD. A study by Nichols et al. highlights the potential for personalized nutritional therapy based on specific microbial signals and diet-related metabolites, which could help target pretreatment optimization and make personalized nutritional therapy for pediatric patients [228]. 

Novel therapeutic foods, such as postbiotics, have been critically reviewed by Lê et al. in the context of managing CD [229]. Postbiotics, which are defined as non-viable bacterial products or metabolic byproducts from probiotic microorganisms, are gaining recognition for their ability to modulate gut microbiota and host responses without the risks associated with administering live bacteria. The review highlights the potential of postbiotics to enhance intestinal epithelial barrier integrity, reduce intestinal permeability, and modulate immune responses through anti-inflammatory mechanisms. For example, microbial metabolites like short-chain fatty acids and bacterial cell wall components such as peptidoglycans and lipoteichoic acids have demonstrated beneficial effects in preclinical models. These compounds can downregulate proinflammatory cytokines and activate regulatory T cells, thereby attenuating inflammation. Lê et al. emphasize that while in vitro and in vivo studies provide promising evidence, clinical data remain limited and heterogeneous. Hence, there is a need for human trials to validate the therapeutic efficacy and safety of postbiotics in CD management [229]. 

Several ongoing trials aim to expand our understanding of dietary interventions in CD, which remain experimental and are not yet part of standard clinical care guidelines. The CORE-IBD initiative is a global, multidisciplinary effort to develop a standardized core outcome set (COS) for randomized controlled trials in IBD, including CD. The main goal of this initiative was to harmonize the selection and reporting of key efficacy and safety endpoints across trials, thereby improving their comparability, quality, and clinical relevance. Core outcome domains identified for CD include patient-reported outcomes (PROs) like abdominal pain and stool frequency, endoscopic assessments, biomarkers such as CRP and FCP, and safety outcomes. The COS is expected to standardize endpoints for drug development and serve as a benchmark to enhance evidence synthesis and reduce heterogeneity in clinical trial design and reporting [230]. 

## Conclusions

Diet is not only a trigger but also a therapeutic tool in CD. From EEN and CDED to the Mediterranean diet, various nutritional approaches can induce remission, sustain disease control, and address malnutrition. Clinicians should prioritize nutrition screening and provide evidence-based dietary guidance tailored to disease phenotype and patient preferences. Collaborative care, involving dietitians and digital health tools, is key to effective implementation. 
